# Health coaching for people with Chronic Obstructive Pulmonary Disease: A commentary on a systematic review

**DOI:** 10.53841/bpstcp.2025.21.1.39

**Published:** 2025-12-22

**Authors:** Claire Niebieski, Karen McNally, Joanna Harrison, James Hill, Nichola Verstraelen

**Affiliations:** a NHS Lancashire and South Cumbria Integrated Care Board, Preston, UK; b Synthesis, Economic Evaluation and Decision Science (SEEDS) group, University of Central Lancashire, Preston, UK; c Lancashire Teaching Hospitals NHS Foundation Trust, Preston, UK

**Keywords:** Chronic Obstructive Pulmonary Disease, Health Coaching, behaviour change, health related quality of life

## Abstract

Chronic Obstructive Pulmonary Disease (COPD) is the third leading cause of mortality worldwide. The symptomatic burden, including breathing difficulties and fatigue, has a profound impact on health-related quality of life and utilisation of health services. The role of health coaching is developing in the health and care system with a focus on improving patient outcomes through personalised goal setting and behaviour change. A systematic review by Long et al. (2019) explored the effectiveness of health coaching for people with COPD on health related, quality of life, mood, physical activity, self-care behaviour and hospital admissions. This commentary critically appraises a systematic review by Long et al. (2019) and considers the findings for COPD care, training and support available for health coaches, coaching psychology for developing research and practice, and the requirements for further research.

## Introduction

Chronic obstructive pulmonary disease (COPD) refers to a group of lung conditions such as emphysema and chronic bronchitis that cause restricted airflow and breathing difficulties (WHO, 2023; NHS, 2023). In the UK, more than 1.2 million people are affected by COPD (Stone et al, 2022) and it remains a common cause of hospital admission (Hopkinson et al, 2019). The overall symptomatic burden for patients has a profound impact on quality of life, and can led to anxiety, depression, fatigue, and a reduced ability to carry out daily tasks or maintain employment (Cook et al, 2018; Martínez-Gestoso, 2022; Miravittles et al, 2017). Moreover, health related quality of life deteriorates with increasing severity of the disease, particularly in those with early onset of COPD (Kharbanda and Anand, 2021). The demands of the disease continue to require attention with prevalence of COPD expected to rise in England, together with direct healthcare costs (McLean et al, 2016).

Health coaching is a supported self-management intervention to help people make informed and conscious choices about their health and become active participants in their care (NHS England, 2023). Health coaches often use behavior change theory and motivational strategies to help patients with the skills required for improving health (Perlman and Abu Dabrh, 2020). In the National Health Service, Health and well-being coaches are personalised care roles which focus on improving patients’ health and lifestyle through goal setting, increasing motivation, self-efficacy, commitment, and behavioural change (NHS England, 2023a).

The role of health coaching is developing in the health and care system as health coaches, and health care professionals adopting health coaching skills, work with people who have long-term conditions, physical and/or mental health conditions (NHS England, 2023a; NHS LSCICB, n.d). Health and wellness coaching is a promising intervention for improving patient outcomes in chronic diseases and lifestyle related disease, improving health behaviours, and reducing health care expenditure (Hale and Giese, 2017; Kreitzer, 2013; Meng 2023; Rethorn and Pettitt, 2019; Prior et al. 2023; Racey 2022; Sforzo et al, 2017).

To determine the effectiveness of health coaching in helping people manage their COPD, improve health related quality of life and reduce healthcare utilisation, Long et al (2019) undertook a systematic review. In this commentary, the findings of the review are subsequently discussed, reviewed through a critical lens, and the implications considered for COPD care, coaching psychologists, and further research.

### Methods of the review by Long et al. 2019

The methodology used to conduct the review by long et al. 2019 is reported in Table 1.

### Results of the review by Long et al. 2019

Of 1578 articles identified in 2016 and 276 searched in 2018, 10 RCTs met the inclusion criteria. Of these, five were conducted in the UK, two in Australia, one in Korea, one in the Netherlands and one in the US. The mean age of participants was 68 and 52% were male. Interventions were delivered by nurses (n=5), a pharmacist (n=1) and a health coach (n=1) and the average number of health coaching sessions was seven. Three RCTs used the Self-Management Programme of Activity, Coping and Education (SPACE) COPD manual. From the results of the quality assessment, most RCTs were generally low risk of bias (69%) or unclear risk (15%).

### Health Related Quality of Life

In the narrative synthesis, four out of ten studies reported a statistically significant improvement to HRQoL and six did not. Of the four RCTs included in the meta-analysis, results showed a significant positive effect for health coaching on the total HRQoL and compared to usual treatment (SMD= -0.69, 95% CI: -1.28 to 0.09). There was a high level of heterogeneity (*I*^*2*^ =93%).

### COPD-related hospital admissions

The narrative synthesis reported that two of seven eligible studies reported a significant reduction for hospital admissions in favour of the intervention group. In meta-analysis, there was a statistically significant reduction in the odds of COPD-related readmission for those who received health coaching compared to those who received usual care in five studies (OR =0.45, 95%CI: 0.30 to 0.67, I^2^=0%). There was no difference in the odds of all-cause hospital admissions between people who received health coaching and those who received usual care in three studies (OR 0.70, 95%CI: 0.41to1.12).

### Physical activity, self-care behaviour, mood, optimal delivery

No meta-analysis was undertaken for these outcomes. Three of eight RCTs reported a significant difference for physical activity in favour of the intervention group with the remaining RCTs showing no significant difference. Three of four RCTs observed significant improvements for self-care behaviour including medication adherence and exercise compliance. One of the three RCTs that investigated the effect of health coaching on mood in people with COPD, observed a significant improvement. For optimal mode of intervention delivery, there was no clear pattern determining one mode of delivery being more effective than another.

### Critical appraisal of the review by Long et al. 2019

Using the AMSTAR-2 critical appraisal tool for systematic reviews (Shea et al, 2017), the review fully achieved six of 16 criteria, three were partially achieved, and seven were not achieved. The seven unmet criteria and the limitations these may present according to The Cochrane handbook (Cochrane Training, 2023) are subsequently described. (1) There was no clear reason given for only including RCTs. Inclusion of non-randomised studies in a systematic review can provide a more complete, real-world picture if carefully assessed. (2) Data extraction was not performed in duplicate. To reduce potential biases introduced by review authors, having more than one person helps to minimise errors. (3) No justification was provided for excluded studies. Demonstrating that all relevant studies are considered helps to answer questions of whether a study was relevant. (4) No sources of funding were reported for included studies. Doing so helps readers assess potential conflicts of interest, maintaining trust in the research process. (5, 6) Although the review did include a risk of bias assessment for the included studies, the review authors did not assess the impact on the meta-analysis or interpret the potential impact of bias on the confidence in the findings. (7) Finally, although justification was provided for not assessing publication bias (a low number of studies), it is important to consider the implications since missing studies can potentially skew the estimate of effects. Overall, the systematic review provides a comprehensive summary of the available data. However, given the limitations of the review outlined, and the considerable heterogeneity of studies (differences between studies being compared), caution should be applied when interpreting the results.

## Discussion

The systematic review by Long et al. (2019) reported positive findings for health-related quality of life and COPD related admissions to hospital however, the evidence was mixed. Recent reviews have explored health coaching within a wider framework of interventions and have reported positive findings. One review identified that multi-modal interventions such as those incorporating self-management, medication support and clinical follow-up showed a trend towards reducing COPD related readmissions (Sharpe et al, 2021). Similarly, a review of improvement interventions including a small number of health coaching interventions found positive outcomes such as a reduction in length of stay, readmissions and use of health resources (MacDonell et al, 2020). Two further systematic reviews have observed that people with COPD may benefit from behaviour change interventions to increase exercise tolerance, physical activity and quality of life (Hanrahan et al, 2024; Hug et al, 2024). In addition, recent individual trials of health coaching, delivered either out of hospital or by lay personnel, have observed positive health outcomes for anxiety, nutritional status, reduced hospitalisation rate, smoking cessation, and engagement with pulmonary speciality care (Noort et al, 2023, Willard-Grace et al, 2023). In areas outside of respiratory, health coaching as a practice has shown some small improvements for adults with chronic pain (Barent-Hepples et al, 2024) and positive psychological change for people with cancer (Vrontara et al, 2023). Studies evaluating the cost-effectiveness of health coaching are limited yet there are some indications that quality of life can be improved, with moderate costs (Oksman et al, 2017).

### Implications for COPD care

The systematic review by Long et al. (2019) and subsequent studies have shown that health coaching and multi-modal interventions may improve health-related quality of life and reduce COPD-related hospital admissions, with additional benefits observed in other areas such as smoking cessation and anxiety reduction, although cost-effectiveness data remains limited. Across Europe, most recommendations for COPD management aim to reduce the severity of current symptoms and the risk of future exacerbations, slowing down the progression of the disease and lowering mortality (Miravitlles et al. 2016). Current UK guidelines emphasise that COPD care should be delivered by a multidisciplinary team that advises people on self-management strategies, education about COPD and identifying/managing those at high risk of exacerbation (NICE, 2019). Although there is no specific mention of health coaching within COPD guidelines, the suggested recommendations could be supported through such an intervention.

Health coaches work across different health settings including primary and secondary care, and can be delivered through a dedicated service or workforce (NHS England, 2023a; NHS England, 2023b). Health coaching skills can also be developed by healthcare professionals within patient consultations and as part of wider workforce development (NHS LSCICB, n.d). In the review by Long et al. (2019), most interventions were delivered by nurses, via in person and telephone consultations. It is difficult to determine the level of health coaching competency as this is not clearly defined. This is important to consider as it has been identified that without a competency framework on which to base health coach training, patient outcomes could be significantly impacted, with more needed to improve regulation and quality (Singh and Kennedy, 2022). Guidance for health care providers on the standards for health and wellbeing coaching, including competencies for the role, capabilities, and guidance on training and support are available are available from both Health Education England (HEE, 2015) and NHS England (NHS England, 2023a). The UK and International Health Coaches Association (UKIHCA) also supports the wide deployment of health coaching for the prevention of ill health and management of long-term chronic disease, and offers coach membership within a defined scope of practice (UKICHA 2023). To further establish the profession, the global wellness institute also recommends the integration of health and wellness coaching boards into primary and secondary healthcare, improving understanding of the professional role within the public and professional arena (O’Connor et al. 2022).

### Coaching psychology for health coaching

As the profession of health coaching continues to develop and grow, the theory and research associated with the psychology of coaching is both applicable and meaningful for developing practice. The psychological professional relationship is embedded in the ‘interpersonal interaction’ of coaching and as such, the study of coaching psychology can enhance health coaching practice and understanding (Passmore & Lai 2019). Several systematic reviews on coaching psychology have previously identified facilitators for a positive coaching outcome, such as the quality of the relationship between client and therapist and motivation to change (Passmore and Lai 2019). More evidence is needed however to determine moderating factors of health coaching interventions such as the number of habits to focus on for successful behaviour change, and effective training for health coaches (Barr et al. 2021). In an expanding field, these unanswered questions require further research to explore the scope and strategies of health coaching and increase the evidence base (Cosgrove & Corrie 2020).

Health coaching has emerged as a patient centred process built on behaviour change theory (Wolever et al. 2013). A such, behavioural analysts have the tools required to address behaviours related to chronic health problems such as lifestyle choices, and are also well positioned to support health coaching practice, to the benefit of both the clients served and the science of behaviour change (Finn and Watson 2017, Normand and Bober 2020). Behavioural science would benefit from increased collaboration with medical professionals and health coaches to support individuals with sustainable behaviour change, target behaviours, and prioritise risk factors for health (Finn & Watson 2017).

### Further research

Given the limitations described in the review by Long et al. 2019, further research in this area should clearly define the intervention to facilitate meta-analysis, and explore heterogeneity. Longer-term outcomes are needed (>12 months) and further exploration of moderating factors including the training level of the provider, delivery (face to face or online/telephone), duration and timing of the intervention. Finally, future studies may benefit from using an accredited patient activation measure at the start and end of the intervention programme. Patient activation is defined as the knowledge, skill and confidence in managing one’s own health and care (The Kings Fund, 2014). Patients who display low levels of activation are typically disengaged and overwhelmed, with poor adherence to goal setting and treatments (Bu and Fancourt, 2021). The use of an evidence-based patient activation measure to apply tailored interventions may improve self-care, and reduce unnecessary healthcare utilisation (Janamian et al, 2022). NHS England provides practical and operational support to organisations looking to implement the Patient Activation Measure in the context of a fully integrated approach to personalised care (NHS England, 2018).

## Conclusion

The authors of Long et al. (2019) describe their findings as ‘non-uniform’ with some studies showing improved outcomes and others not, yet they conclude that health coaching for COPD may be a ‘candidate intervention to improve these important outcomes in COPD’. The wider literature suggests positive results from health coaching interventions yet more clearly defined research is needed to confirm this, particularly in the longer-term. The coaching psychology field is well placed to support the research, training and service development needs of health coaching, and collaboration with health care providers would be beneficial. Those working in health coaching should consult the NHS England guidance available and consider the use of a patient activation measure to inform tailored interventions and personalised care.

## Figures and Tables

**Table 1:** Methodology of the review by Long et al. 2019.

Search Strategy	A comprehensive search of electronic databases up to August 2018.
Inclusion Criteria	- Randomised Controlled Trials (RCTs) of health coaching interventions for adults with COPD. - Interventions that included evidence of goal setting, motivational interviewing, and COPD-related health education. - Interventions delivered on an individual basis, by a health care professional, and over a minimum of two sessions. - Interventions compared to either usual treatment, wait-list control or no intervention control group. - The primary outcome measure was a validated, self-report measure of general quality of life and/or disease specific Health Related Quality of life (HRQoL).
Screening Process	Screening of articles was completed by one author and a subset of potentially eligible articles up to 2016 was checked by a second reviewer. Inter-rater reliability was calculated with a score of 91%.
Data Extraction	Data extraction was undertaken by one author.
Quality of Evidence	Quality appraisal was undertaken using the Cochrane Risk of Bias tool by two authors independently and with disagreements resolved by a third reviewer.
Data Analysis	Data were analysed by narrative synthesis and where possible, a meta-analysis using a random effects model. Effect sizes were calculated using Standardised Mean Differences (SMDs) and 95% Confidence Intervals (CIs). Odds ratios (OR) and 95% CIs were calculated for reported hospital admissions. Where multiple follow-up periods were stated, the data collection nearest to 6 months was utilised for consistency across studies. Heterogeneity of studies was reported using the chi-squared test and *I^2^* statistic.

## Key Points

▪ Health coaching supports individuals with behavioural changes related to their health, through personalised strategies such as goal setting and increased self-efficacy.
▪ There is some evidence to suggest that health coaching for COPD has a positive effect on health-related quality of life, and for COPD related admissions to hospital.
▪ Guidance, training and support is available for health coaching professionals to ensure clear standards and competencies.
▪ Coaching psychology can support and develop the needs of health coaching practice through theory and research.
▪ Longer-term research is needed to build the evidence base, and should include moderating factors such as the mode of delivery and training level of the provider.
